# Insulin Signalling‐Inducible IFITM1 Promotes Multiple Myeloma Progression and Bortezomib Resistance

**DOI:** 10.1111/jcmm.71183

**Published:** 2026-06-12

**Authors:** Ji‐Young Lim, Yeojin Kim, Sung‐Soo Park, Jungyeon Lee, Chang‐Ki Min

**Affiliations:** ^1^ Department of Internal Medicine, Seoul St. Mary's Hospital The Catholic University of Korea Seoul Korea

**Keywords:** autologous stem cell transplantation, bortezomib, insulin receptor, interferon‐induced transmembrane protein 1, multiple myeloma

## Abstract

Insulin substantially promotes the growth of malignant cells that overexpress the insulin receptor (INSR), and insulin excess has been recognised as a cancer‐promoting factor in patients. Interferon‐induced transmembrane protein 1 (IFITM1) is also overexpressed in various cancers. In this study, we investigate the association between insulin signalling‐induced IFITM1 expression and multiple myeloma (MM) aggressiveness. We observed that expression of both INSR and IFITM1 was significantly elevated in symptomatic MM patients compared with those with monoclonal gammopathy of undetermined significance (MGUS) and smouldering MM (SMM). Notably, IFITM1—but not INSR—expression correlated with prognosis following autologous stem cell transplantation and bortezomib‐based induction therapy. Further analysis revealed that IFITM1 expression in bone marrow plasma cells was associated with the concentrations of insulin and insulin‐like growth factor 2 (IGF‐II) in the bone marrow microenvironment. Insulin and IGF‐II enhanced MM cell proliferation through IFITM1 upregulation, whereas suppression of IFITM1 abrogated the proliferative effects of these ligands. Moreover, insulin and IGF‐II attenuated apoptosis and the inhibition of cell migration induced by the proteasome inhibitors (PIs) bortezomib and carfilzomib, and these effects were reversed by IFITM1 knockdown. The ability of insulin to reduce bortezomib‐induced apoptosis and G2/M phase cell cycle arrest was likewise dependent on IFITM1 expression. Collectively, these findings suggest that insulin‐induced IFITM1 plays a pivotal role in MM progression and resistance to bortezomib, highlighting IFITM1 as a potential prognostic biomarker and therapeutic target.

## 1 Introduction

Multiple myeloma (MM) is a haematological malignancy characterised by clonal proliferation of abnormal plasma cells (PCs) in the bone marrow (BM), leading to haematopoietic failure, lytic bone lesions, and kidney damage. Despite the significant advancements provided by novel chemotherapeutic agents and cell therapy over the past two decades, MM is incurable. Patients may have temporary remission, but this will inevitably be followed by relapse due to the development of treatment resistance [1, 2]. **S**urvival and proliferation of malignant PCs are regulated by complex interactions in the cancer microenvironment. These interactions include crosstalk between various cell types of the immune system, and cytokines and chemokines have important roles. Understanding these crosstalk and molecular interactions may offer opportunities for therapeutic intervention [3, 4]. Therapies targeting the microenvironment can potentially disrupt supportive signals for cancer cells and impede disease progression.

Insulin, a hormonal growth factor produced in the pancreas, has a potent stimulatory effect on the growth of malignant cells, particularly those that overexpress the insulin receptor (INSR) [5, 6]. Alternative splicing during INSR transcription produces INSR‐A or INSR‐B isoforms, and INSR‐A is notably overexpressed in cancer [7]. Therefore, insulin and INSR may play an essential role in the proliferation and growth of cancer cells [8, 9]; however, unlike the role of insulin‐like growth factor (IGF), the roles of insulin and INSR in MM have not been extensively studied [10, 11]. While the IGF‐I/IGFIR axis has been intensively investigated in MM for its mitogenic and anti‐apoptotic effects [10, 11], the INSR pathway remains comparatively underexplored in MM. Importantly, INSR‐A, the isoform enriched in malignant cells, can drive proliferative signalling [7, 8, 9], and INSR is activated not only by insulin but also by IGF‐II present in the BM microenvironment, pointing to a biologically distinct route of receptor engagement from canonical IGF1R signalling [5, 6, 7, 8, 9, 11]. On this basis, we focused our study on INSR activation by its physiological ligands to define INSR‐specific contributions to myeloma growth and drug response.

Interferon‐induced transmembrane protein 1 (IFITM1), B cell co‐receptor Leu 13, is an IFITM family member [12]. IFITM1 functions primarily in immune response signalling and controls cell proliferation, promotes homotypic cell adhesion, and prevents viral infection [13]. Recent studies have shown that IFITM1 is overexpressed in numerous human cancers and plays a vital role in tumorigenesis. IFITM1 is overexpressed in several types of cancers, including lung, cervical, ovarian, colorectal, and breast cancers. In these cancers, IFITM1 overexpression positively correlates with tumour progression and increased invasiveness [14, 15, 16, 17, 18]. Notably, insulin/INSR signalling has been reported to upregulate IFITM family expression [19], raising the possibility that INSR‐driven IFITM1 induction could couple metabolic/growth signals to survival programs in MM.

Here, we investigate the clinical and biological relevance of the INSR‐IFITM1 axis in MM by integrating patient‐derived data with mechanistic experiments. We show that INSR ligands induce IFITM1, that IFITM1 is required for INSR‐dependent growth and proteasome inhibitor resistance, and that higher IFITM1 expression associates with adverse clinical outcomes, implicating IFITM1 as both a prognostic maker and a therapeutic target in INSR‐active myeloma.

## 2 Materials and Methods

### 2.1 Patients and Transplant Procedures

BM samples were obtained from 19 patients diagnosed with monoclonal gammopathy of undetermined significance (MGUS), 19 patients with smouldering MM (SMM), and 92 patients with symptomatic MM. A total of 82 consecutive patients with MM who underwent first‐line autologous stem cell transplantation (ASCT) following bortezomib (BTZ)‐based induction treatment at our institution between January 2015 and December 2020 were enrolled for this analysis. Cytogenetic findings and international staging system (ISS) determinations were obtained from the diagnostic data. High‐risk cytogenetic abnormalities were defined as the presence of del(17p), t(4;14), and/or t(14;16). ASCT was performed after achieving more than a partial response. General ASCT procedures were performed as described previously [20]. Briefly, all patients were mobilised with cyclophosphamide (3 g/m^2^ total) for two days and then treated once daily with subcutaneous G‐CSF (lenograstim; JW Pharmaceutical, Seoul, Korea) at 10 μg/kg/day. Patient conditioning was usually achieved with 100 mg/m^2^ of melphalan for two days, but patients with serum creatinine > 2.0 mg/dL or on haemodialysis received 70 mg/m^2^ for two days. Written informed consent was obtained from each patient before participation in this study. The current study was approved by the institutional review board of the Catholic University of Korea, College of Medicine (KC12SISE0594).

### 2.2 BM Sample Collection and Isolation of Plasma Cells (PCs)

BM mononuclear cells were freshly isolated from the whole BM (10 mL). BMPCs were purified using CD138‐positive separation microbeads (Miltenyi Biotec, 130–051‐301).

### 2.3 Quantitative Reverse Transcription (qRT)‐PCR Analysis

One microgram of total RNA was reverse transcribed into cDNA. Quantitative assessment of target mRNA levels was performed by real‐time PCR with a CFX96 Real‐Time PCR Detection System (Bio‐Rad). The mRNA quantity was calculated using the 2–^ΔΔCt^ method, and the level of GAPDH was used to normalise total RNA quantities (ref). Target‐specific primers and sequences were:

INSR Forward: CCCCAGAAAAACCTCTTCAGG.

INSR Reverse: GTCACATTCCCAACATCGCC.

IFITM1 Forward: GGTCCCTGTTCAACACCCTC.

IFITM1 Reverse: CTGTCACAGAGCCGAATACC.

GAPDH Forward: ACCCACTCCACCTTTGA.

GAPDH Reverse: CATACCAGGAAATGAGCTTGACAA.

### 2.4 Cytokine Measurements Using ELISA

Insulin (R and D Systems, DINS00) and insulin‐like growth factor 2 (IGF‐II, R and D Systems DG200) concentrations were measured in the patient's BM plasma by ELISA. The assays were performed according to the manufacturer's protocol. The ELISA plates were analysed using a microplate reader (VersaMAx, Molecular Devices).

### 2.5 Human Myeloma Cell Line (HMCL) Culture Conditions and Reagents

RPMI8226 and U266 cells were purchased from ATCC (Manassas, VA, USA). These cells were cultured in RPMI1640 medium supplemented with 10% FBS and 1% penicillin–streptomycin‐glutamine solution (Invitrogen). Recombinant human insulin and IGF‐II were purchased from PepproTech (#10–365, #100–12); the proteasome inhibitors (PIs), such as BTZ and carfilzomib (CFZ), were from Selleckchem (S1013, S2853).

### 2.6 CFSE Cell Proliferation Assay

PRMI8226 and U266 cells were stained with 2.5 μM CellTrace CFSE (Thermo Fisher, C34554) in 2.5% FBS in PBS at 37°C for 10 min, and then were washed with serum‐free medium. Cells were analysed immediately by flow cytometer (zero time point), and the cells were seeded in 96‐well tissue culture plates with insulin and IGF‐II and incubated at 37°C. Proliferation of HMCLs was analysed using LSRII Fortessa (BD Pharmigen) and Flowjo software.

### 2.7 Apoptosis Assay

RPMI8226 and U266 cells were cultured with or without insulin and IGF‐II. The co‐cultured cells were incubated with or without 10 nM BTZ and CFZ. After incubation for 48 h, the cells were harvested, stained with annexin V‐APC (BD Biosciences, 561,012) and propidium iodide (PI, BD Biosciences, 556,463), and examined by flow cytometry. Data obtained from the LSRII Fortessa (BD Pharmingen) were analysed using Flowjo software.

### 2.8 Western Blot Analysis

For analysis, 20 ug of total extracts were separated by SDS‐PAGE and transferred onto nitrocellulose membranes (Whatman). The blots were incubated with antibodies against p‐mTOR (1:1000, cell signalling, #5536), mTOR (1:1000, cell signalling, #2983), p‐Akt (1:1000, cell signalling, #9271), Akt (1:1000, cell signalling, #4691), p‐AMPK (1:1000, cell signalling, #2535), AMPK (1:1000, cell signalling, #2532), p53 (1:1000, Abcam, ab26), p21 (1:2000, Abcam, ab109520), bax (1:500, Abcam, ab7977), caspase 3 (1:2000, Cell Signalling Technology, 9662), caspase 9 (1:2000, Cell Signalling Technology, 9502), and IFITM1 (1:2000, Abcam, ab233545) using β‐actin (1:5000, Abcam, ab75186) as a loading control. The signals were detected using the ECL detection system (Bio‐Rad). Western blot densitometry was analysed using ImageJ software.

### 2.9 Migration Assay

Cell migration was detected using a transwell system. Briefly, 1 × 10^5^ RPMI8226 and U266 cells in 100 ul RPMI1640 medium containing 0.1% FBS were loaded on top of the filter membranes of 24‐well transwell plates (3 μm pore size; Corning). Insulin or IGF‐II was resuspended in RPMI1640 medium with or without BTZ and CFZ and loaded on the bottom of the membranes. After 24 h of incubation, the transwell insert was removed from the plate, and the media and non‐migrating cells were carefully removed from the top of the membrane using a cotton‐tipped applicator to avoid damage to the membrane. Next, the transwell insert and migrated cells were placed in 4% PFA for 15 min for cell fixation. After fixation, the cells were washed with PBS for 5 min. The transwell insert was stained by placement in a 0.1% crystal violet solution for 30 min, and after removing excess crystal violet, the migrated cells were imaged under an inverted microscope (x200). Five independent experiments were performed for each condition.

### 2.10 siRNA Transfection

The RPMI8226 and U266 cells were seeded into six‐well plates at 2 × 10^5^ cells/well. siRNA was purchased from Ambion Applied Biosystems and used according to the manufacturer's instructions. Suppression of IFITM1 was accomplished using si‐IFITM1 #1 (ID #s2299) and si‐IFITM1 #2 (ID #s2300). Transfection was performed using Lipofectamine 2000 (Thermo Scientific) with siRNA constructs and scrambled negative control siRNAs.

### 2.11 Cell Viability Assay

Measurement of cell proliferation was achieved using the CellTiter 96 AQ_ueous_ One Solution Cell Proliferation Assay (MTS) kit (Promega, G3582). The siRNA‐transfected RPMI8226 and U266 cells were seeded in 96‐well tissue culture plates at 3 × 10^4^ cells/well with or without insulin and IGF‐II and incubated at 37°C for 48 h. After 20 h of recombinant protein exposure, 10 μL of MTS reagent was added to each well, and the cells were incubated at 37°C for 4 h. Absorbance was detected at 490 nm with a microplate reader (VersaMAx, Molecular Devices).

### 2.12 Cell Cycle Analysis

RPMI8226 and U266 cells were cultured with or without insulin. The co‐cultured cells were then incubated with or without 10 nM BTZ. After incubation for 48 h, the cells were harvested and washed with PBS. Cold 70% ethanol was used for cell fixation. After 2 h, the cells were washed in PBS and resuspended in PBS containing PI (50 ug/ml), RNase A (100 ug/ml), and 0.1% Triton X‐100 overnight at 4°C. Data obtained from the LSRII Fortessa (BD Biosciences) were analysed using Flowjo software.

### 2.13 Definitions and Statistical Analysis

Progression‐free survival (PFS) was calculated as the time from BTZ‐containing induction treatment and ASCT to disease progression. Deaths due to causes other than progression were censored. Patients lost to follow‐up were also censored at the date of last contact. Survival curves were plotted according to the Kaplan–Meier method, and the log‐rank test was used to assess potential prognostic factors. An independent sample *t*‐test and one‐way ANOVA were used to compare the statistical significance of the differences between two or more samples. Two‐tailed *p*‐values < 0.05 were considered statistically significant. The correlation analysis was assessed using the Spearman correlation coefficient and chi‐square test. Statistical analyses were performed by GraphPad Prism Software.

## 3 Results

### 3.1 INSR And IFITM1 Expression in Primary BMPCs

**FIGURE 1:**
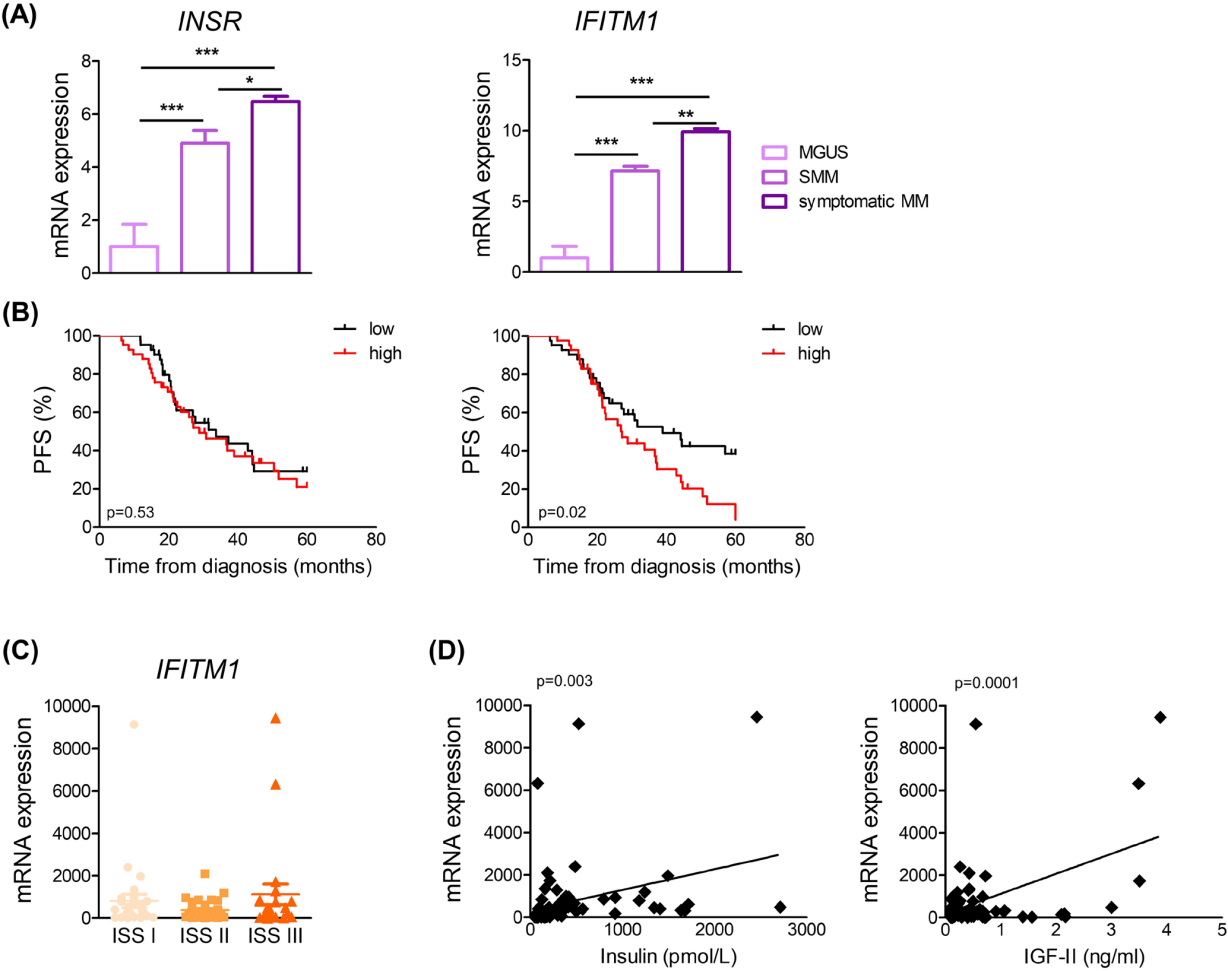
INSR and IFITM1 expression in BM plasma cells (PCs) and correlation with disease aggressiveness. (A) Expression of INSR and IFITM1 mRNA was evaluated by real‐time PCR using isolated BM CD138+ PCs from MGUS (*n* = 19), SMM (*n* = 19), and symptomatic MM (*n* = 92) patients at the time of diagnosis. The data are presented as mean ± SEM. *p < 0.05, **p < 0.01, ***p < 0.001 (one‐way ANOVA). (B) Kaplan–Meier estimated curves represent the probability of progression‐free survival (PFS) of patients with MM who underwent ASCT (*n* = 82) according to the level of INSR and IFITM1 expression (low group is in black; and high group is in red). Survival curves were plotted according to the Kaplan–Meier method, and the log‐rank test was used to assess potential prognostic factors. (C) IFITM1 expression in BM PCs from ISS stage I (*n* = 30), stage II (*n* = 41), and stage III MM (*n* = 22) patients. (D) Correlation analysis between IFITM1 expression and levels of insulin receptor ligands insulin and IGF‐II in BM plasma.

We compared INSR and IFITM1 mRNA expression in primary BMPCs from MGUS (*n* = 19), SMM (*n* = 19), and symptomatic MM (*n* = 92) patients at the time of diagnosis. Figure 1A shows that the mRNA expression levels of INSR and IFITM1 in isolated BMPCs significantly increased during the transition from MGUS to symptomatic MM. Next, we evaluated the correlation of each mRNA expression level and ASCT outcome. The patients were grouped according to the median value of mRNA expression level. The association of INSR with the five‐year PFS was analysed and showed no difference between the high and low groups (28.8% vs. 33.7%, *p* = 0.53) (Figure 1B, left). In contrast, the five‐year PFS was significantly lower in the high IFITM1 group compared with the low IFITM1 group (39% vs. 27.2%, *p* = 0.02) (Figure 1B, right). IFITM1 mRNA expression, however, did not correlate with the ISS (Figure 1C). To further investigate the correlation between IFITM1 mRNA expression and INSR ligand levels, we analysed patient BM plasma by ELISA. Higher levels of IFITM1 mRNA were observed in patients with elevated insulin and IGF‐II concentrations, suggesting a potential role for these INSR ligands in inducing IFITM1 expression (Figure 1D).

### 3.2 Proliferative Effects of INSR Ligands on HMCLs Require the Presence of IFITM1

**FIGURE 2:**
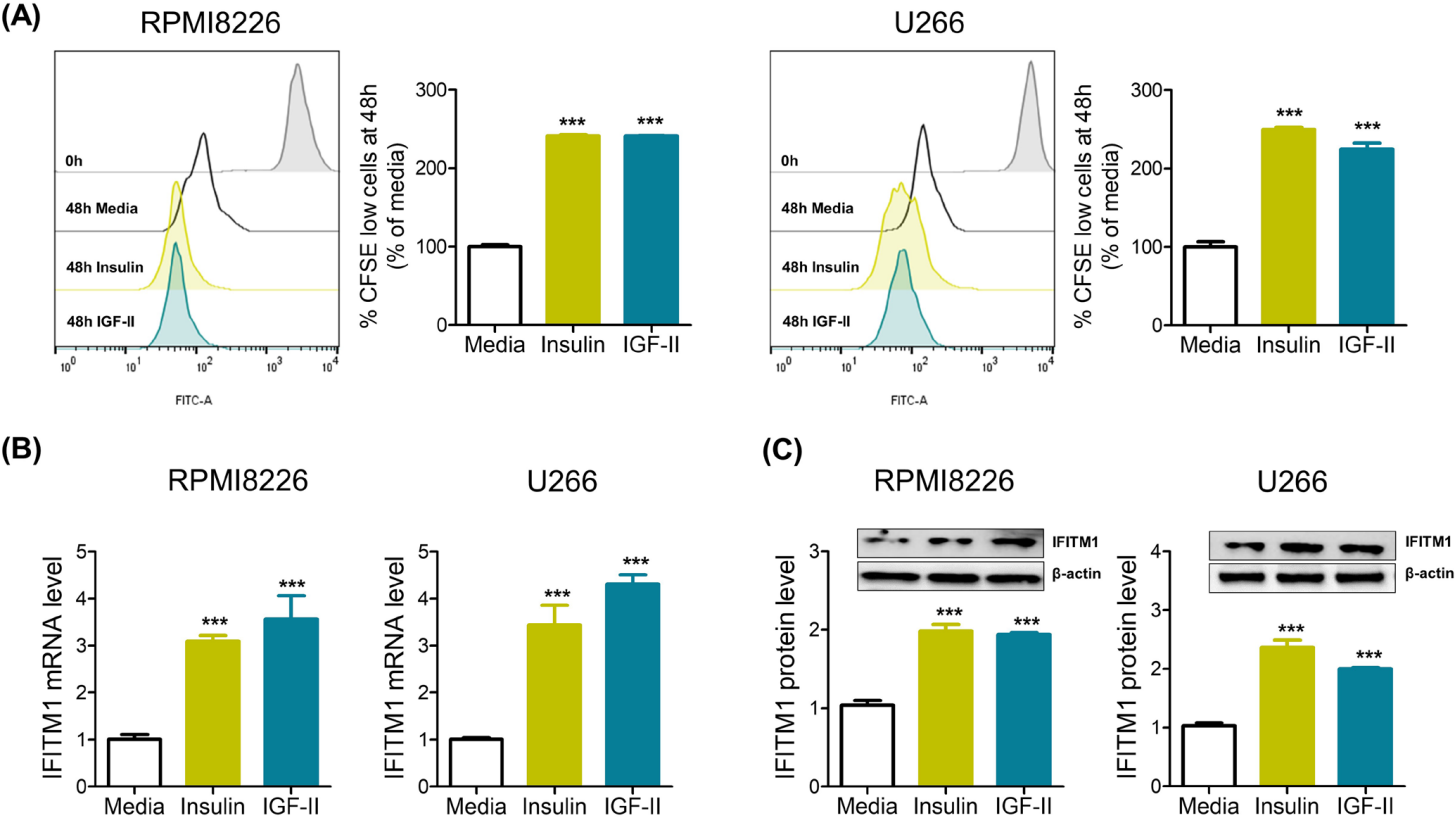
INSR ligand‐induced IFITM1 expression regulates the proliferation of human myeloma cell lines (HMCLs). (A) RPMI8226 and U266 cells were cultured with or without insulin (100 ng/mL) and IGFII (100 ng/mL) for 48 h. Cell proliferation was quantified using the CFSE dilution assay and analysed by flow cytometry. Representative histograms and quantification of divided cells are shown. The data are presented as mean ± SEM of three independent experiments. (B, C) The effect of INSR ligands on IFITM1 expression was analysed by qPCR (B) and western blot (C) in RPMI8226 and U266 cells. The data are presented as mean ± SEM of three independent experiments. ***p < 0.001 (one‐way ANOVA).

**FIGURE 3:**
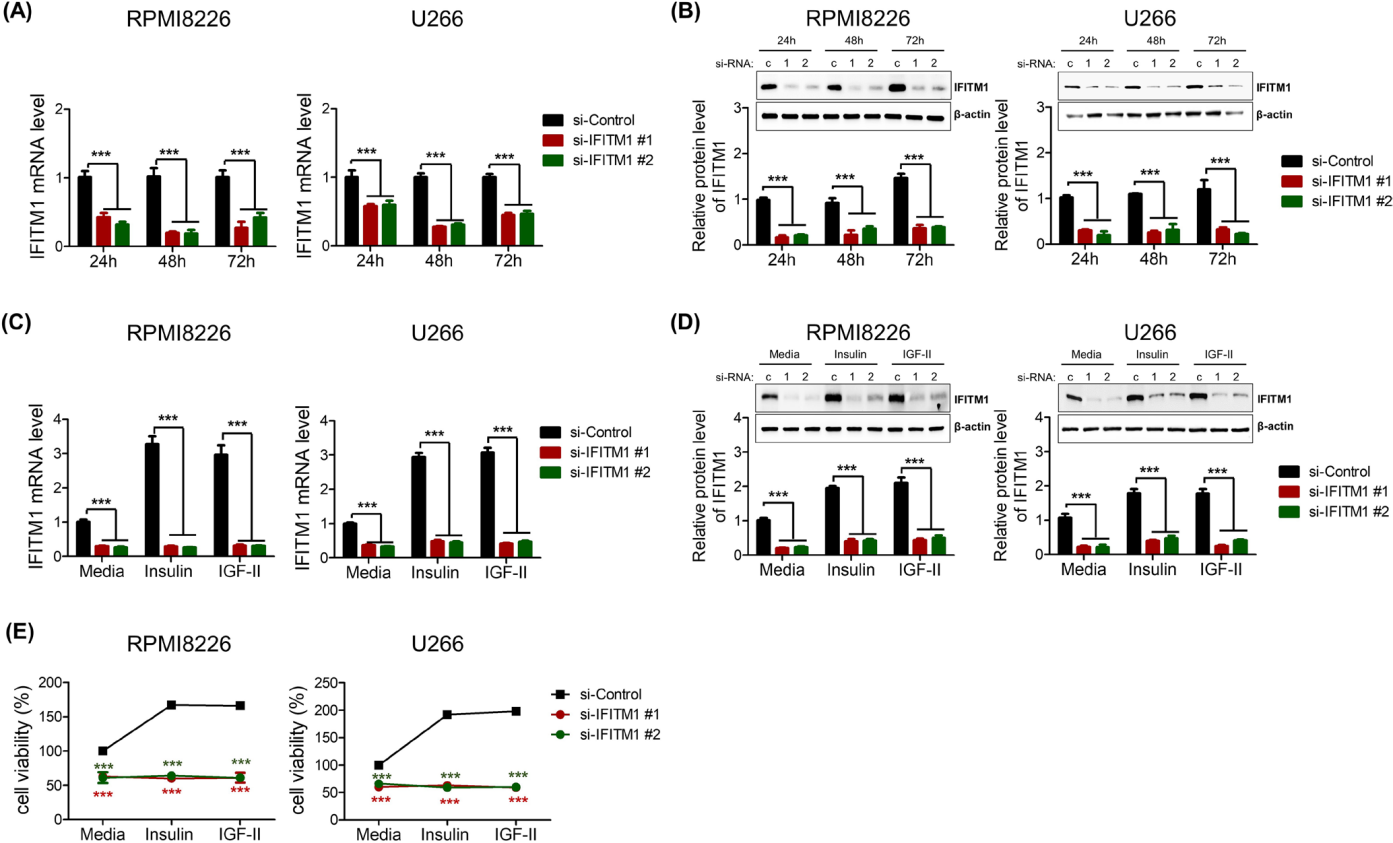
IFITM1 is essential for the proliferation of multiple myeloma cells by the INSR signalling pathway. The efficiency of two siRNA sequences against IFITM1 was evaluated by qPCR (A) and western blotting (B). The effect of INSR ligands on IFITM1 expression in control or si‐IFITM1‐transfected RPMI8226 and U266 cells was confirmed by qPCR (C) and western blotting (D). (E) Proliferation of control or si‐IFITM1‐transfected RPMI8226 and U266 cells in response to INSR ligands was evaluated using the MTS assay. The data are presented as mean ± SEM of three independent experiments. ***p < 0.001 (one‐way ANOVA).

The treatment with INSR ligands, specifically insulin and IGF‐II, significantly enhanced the proliferation of RPMI8226 and U266 cells (Figure 2A). In addition, treatment of RPMI8226 and U266 cells with insulin and IGF‐II significantly increased the mRNA (Figure 2B) and protein (Figure 2C) levels of IFITM1. To investigate the role of IFITM1 in the context of MM progression, we explored the influence of the absence of IFITM1 on the regulation of MM cells. Transfection with siRNA IFITM1 sequences significantly reduced IFITM1 mRNA levels compared to control cells transfected with scrambled siRNA in a time‐dependent manner (Figure 3A). Similarly, Figure 3B shows a time‐dependent decrease in IFITM1 protein expression in siRNA‐IFITM1‐transfected cells compared to control cells.

To investigate the interplay between INSR signalling and IFITM1 expression in MM cell growth, IFITM1 expression was manipulated in RPMI8226 and U266 cells through siRNA transfection, and RPMI8226 and U266 cells were subsequently treated with INSR ligands. Our results demonstrated that INSR ligands induced increased IFITM1 mRNA (Figure 3C) and protein levels (Figure 3D); these increases were abrogated in cells transfected with si‐IFITM1. Furthermore, the suppression of IFITM1 attenuated the proliferative response of MM cells to INSR ligands (Figure 3E). These data indicate that IFITM1 is necessary for INSR‐mediated cell proliferation.

### 3.3 INSR Ligands Mitigate Proteasome Inhibitors‐Induced Apoptosis and Cell Migration

**FIGURE 4:**
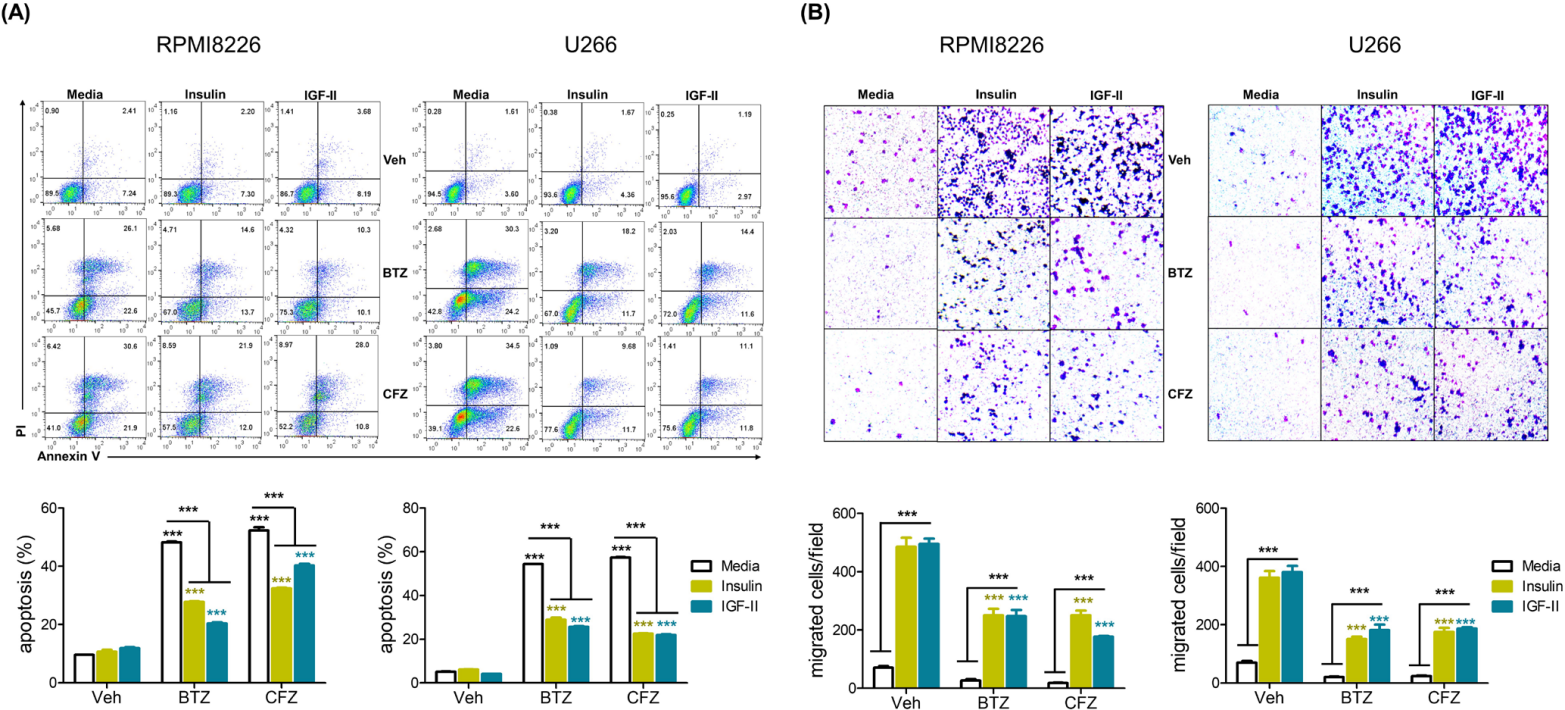
INSR ligands‐induced IFITM1 expression regulates multiple myeloma cell proteasome inhibitors (PIs)‐induced apoptosis and cell migration. (A) The effect of INSR ligands on PIs‐induced apoptosis was tested. The Annexin V and PI levels of RPMI8226 and U266 cells incubated with BTZ and CFZ in the presence or absence of INSR ligands. The data are presented as mean ± SEM of three independent experiments. (B) Migration of RPMI8226 and U266 cells was examined using a transwell assay with or without BTZ and CFZ. INSR ligands were used as chemoattractants. The data are presented as mean ± SEM of three independent experiments. ***p < 0.001 (one‐way ANOVA).

Next, we explored the effect of insulin and IGF‐II on the treatment response of MM cell exposure to proteasome inhibitors, particularly concerning their ability to resist apoptosis and inhibit migration induced by BTZ and CFZ. INSR ligands not only attenuated cell apoptosis induced by BTZ and CFZ in RPMI8226 and U266 cells (Figure 4A) but also enhanced cell migration inhibited by these inhibitors (Figure 4B). These results suggest that insulin and IGF‐II, through their interaction with INSR, can undermine the effectiveness of BTZ and CFZ by both protecting MM cells from apoptosis and allowing their continued migration.

To further clarify whether these effects reflected a transient delay or a persistent cytoprotective response, a time‐course analysis was conducted at 24, 48, and 72 h after BTZ treatment (Figure S1). Insulin and IGF‐II consistently reduced apoptotic cells throughout the observation period. This reduction persisted up to 72 h, indicating sustained protection against PIs‐induced cytotoxicity rather than a temporal delay. Similarly, migration assays performed at 6, 12, and 24 h demonstrated that both ligands continuously counteracted BTZ‐induced inhibition of cell migration (Figure S2). Collectively, these results establish that insulin and IGF‐II exert durable cytoprotective and pro‐migratory effects on MM cells under proteasome inhibition, reinforcing the contribution of INSR signalling in therapeutic resistance and disease progression.

### 3.4 IFITM1 Mediates Insulin‐Dependent Activation of mTOR and Akt Signalling in MM Cells

**FIGURE 5:**
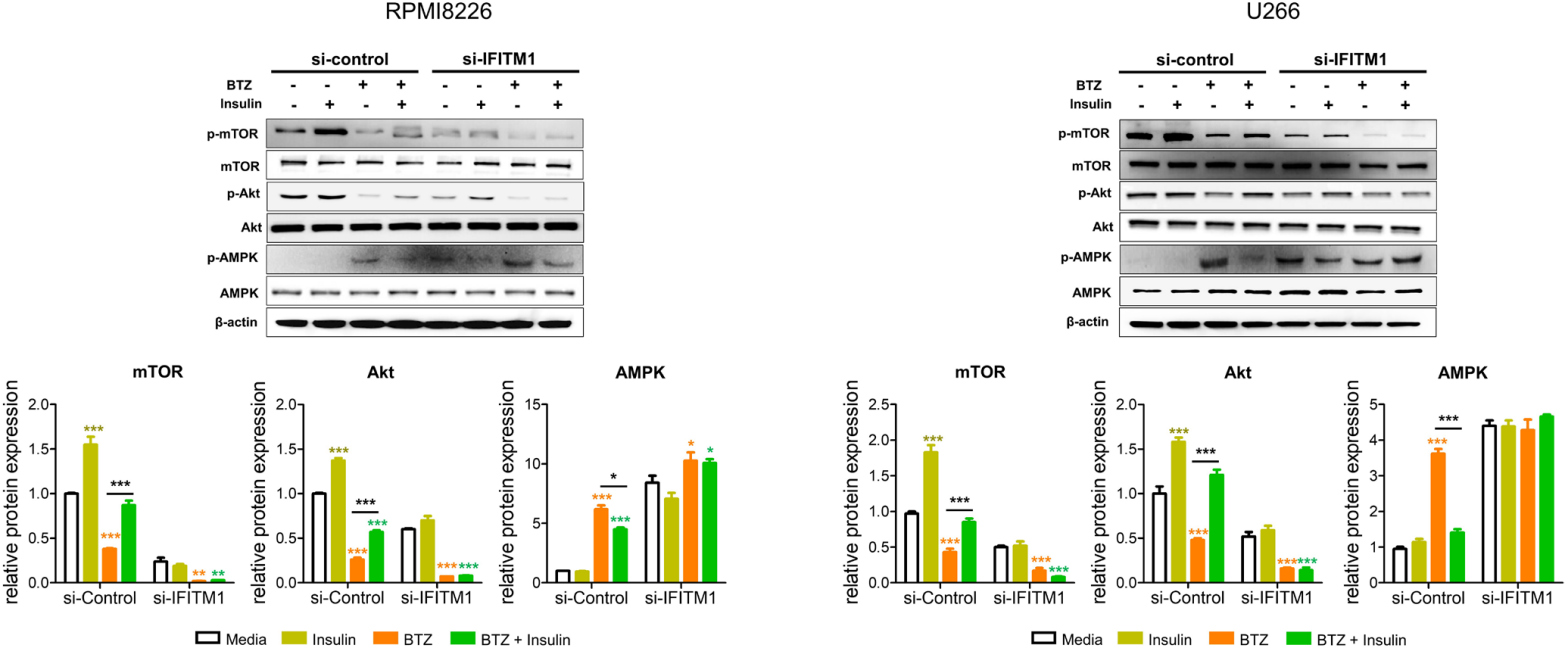
IFITM1 silencing disrupts INSR signalling‐induced mTOR–Akt activation. Western blot analyses of RPMI8226 and U266 cells transfected with si‐control or si‐IFITM1 and treated with or without insulin and BTZ. Phosphorylated and total levels of mTOR, Akt, and AMPK were examined. β‐actin was used as a control. The data are presented as mean ± SEM of three independent experiments. ***p < 0.001 (one‐way ANOVA).

To elucidate the mechanistic link between INSR signalling and IFITM1 function, we analysed the activation status of key downstream signalling molecules involved in metabolic regulation, including mTOR, Akt, and AMPK, in RPMI8226 and U266 cells. In siRNA‐control cells, insulin treatment markedly increased the phosphorylation of mTOR and Akt, consistent with the activation of anabolic and pro‐survival signalling pathways. In contrast, siRNA‐IFITM1 cells significantly attenuated basal mTOR and Akt phosphorylation and enhanced AMPK activation, indicating a metabolic shift toward catabolic stress signalling. Notably, in IFITM1‐suppressed cells, insulin failed to restore mTOR and Akt activation, demonstrating that IFITM1 is required for insulin responsiveness in MM cells. Upon BTZ treatment, phosphorylation of more and Akt was markedly reduced, whereas AMPK phosphorylation increased, consistent with the induction of a stress and apoptotic state. Insulin co‐treatment partially reversed BTZ‐induced inhibition of mTOR and Akt and reduced AMPK phosphorylation, reflecting a cytoprotective signalling response. However, this effect was completely abolished in IFITM1 KD cells (Figure 5). Collectively, these results demonstrate that IFITM1 is essential for insulin‐mediated activation of mTOR and Akt signalling, supporting metabolic adaptation and survival under proteasome inhibitor stress. These findings establish a mechanistic basis for the cytoprotective role of the insulin‐INSR‐IFITM1 axis in MM.

### 3.5 Loss of IFITM1 Disrupts the Resistance Induced by Insulin Following Treatment With BTZ

**FIGURE 6:**
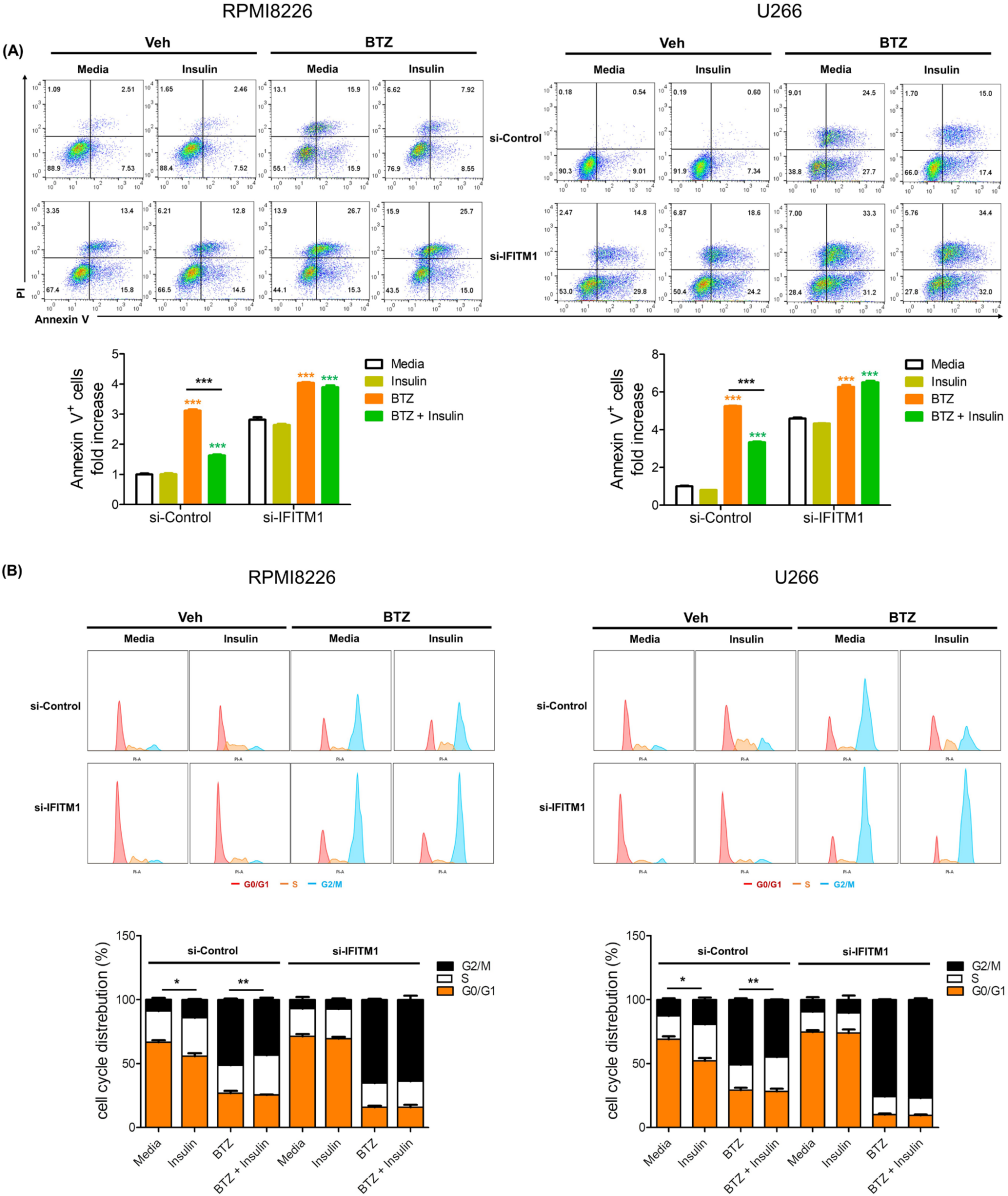
Loss of IFITM1 increased INSR signalling‐induced and BTZ‐resistant RPMI8226 and U266 cells apoptosis and G2/M phase arrest. (A) The effect of insulin on BTZ‐induced apoptosis in control or si‐IFITM1‐transfected RPMI8226 and U266 cells was confirmed by FACS analysis. The data are presented as mean ± SEM of three independent experiments. (B) Effect of insulin and BTZ on cell cycle distribution. Control or si‐IFITM1‐transfected RPMI8226 and U266 cells were cultured with or without insulin and BTZ. Then, cell cycle profile analysis was conducted from the flow cytometry results. Cell populations in the G0/G1, S, and G2/M phases are presented as the mean percentage of total cells. The data are presented as mean ± SEM of three independent experiments. ***p < 0.001 (one‐way ANOVA).

To assess whether the absence of IFITM1 eliminated the resistance induced by the insulin signalling after BTZ treatment, RPMI8226 and U266 cells transfected with siRNA‐IFITM1 were subsequently treated with insulin and/or BTZ to investigate the impact of IFITM1 suppression on MM cell apoptosis. Our results demonstrated that insulin treatment decreased BTZ‐induced siRNA‐control RPMI8226 and U266 cell apoptosis, but insulin could not reduce the apoptosis induced by BTZ treatment in siRNA‐IFITM1 RPMI8226 and U266 cells (Figure 6A). To assess the underlying mechanisms, we conducted cell cycle analysis. In IFITM1‐intact RPMI8226 and U266 cells, insulin treatment induced progression to the S phase; siRNA‐IFITM1 RPMI8226 and U266 cells did not exhibit this S phase induction when treated with insulin. Furthermore, in IFITM1‐intact RPMI8226 and U266 cells, BTZ treatment induced G2/M phase arrest, but this BTZ‐induced cell cycle arrest was attenuated when the cells were treated with insulin. However, in IFITM1‐suppressed RPMI8226 and U266 cells, the G2/M phase arrest was maintained even with BTZ and insulin treatment (Figure 6B).

**FIGURE 7:**
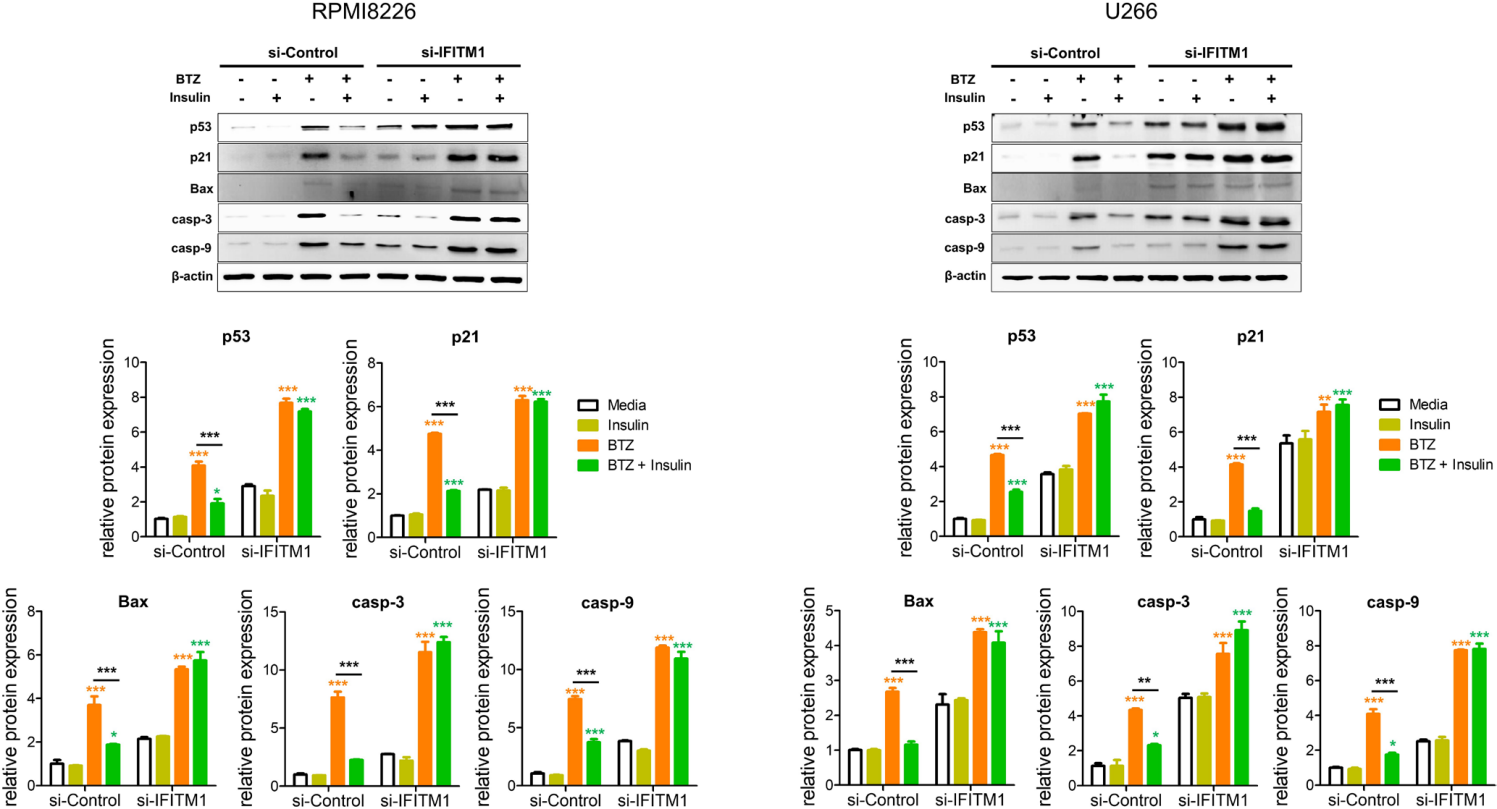
IFITM1 Silencing enhances INSR signalling‐induced and BTZ‐resistant RPMI8226 and U266 cells apoptosis via the p53 signalling pathway. Control or si‐IFITM1‐transfected RPMI8226 and U266 cells were treated with or without insulin and BTZ. Western blot analysis was performed on apoptotic proteins. β‐Actin was used as a control. The data are presented as mean ± SEM of three independent experiments. ***p < 0.001 (one‐way ANOVA).

We investigated the expression levels of key apoptotic proteins. Our findings revealed that BTZ treatment upregulated the expression of pro‐apoptotic proteins such as p53, p21, Bax, caspase 3, and caspase 9. Conversely, insulin treatment downregulated pro‐apoptotic protein expression, counteracting the effect of BTZ. However, the absence of IFITM1 mitigated the downregulated pro‐apoptotic proteins in the presence of BTZ and insulin, suggesting that IFITM1 may play a role in regulating their expression (Figure 7). These results suggest that IFITM1 is crucial for the resistance that insulin confers to MM cells when these cells are treated with BTZ.

## 4 Discussion

Our study explored the possibility of a link between insulin signalling and the expression of IFITM1 in terms of the aggressiveness and treatment resistance of MM. Insulin signalling is a complex network of molecular pathways triggered by the hormone insulin. Insulin signalling is traditionally recognised for its metabolic role, yet numerous studies have shown that the same pathway exerts strong mitogenic and anti‐apoptotic effects in malignant cells [21, 22]. Notably, the INSR can act as a dual‐function receptor that, depending on its activation context and isoform composition, mediates both metabolic and growth‐promoting functions [7, 8, 9].

IFITM1 is also overexpressed in various cancer types [23], and its upregulation has been associated with tumour proliferation, invasion, and resistance to apoptosis. Therefore, the study specifically focused on the expression of IFITM1 induced by insulin signalling in MM. INSR and IFITM1 expression in malignant PCs was increased as the disease advanced, but only IFITM1 had significance in predicting disease prognosis. We showed that the molecular interactions involving INSR ligands and the expression of IFITM1 could contribute to the MM cell growth and migration in terms of BTZ treatment resistance. Of note, IFITM1 suppression reversed the protective effects of insulin signalling on MM cells, implying that IFITM1 plays a role in modulating cellular response to insulin and BTZ treatment. Also, the insulin‐modulating effect on cell cycle arrest and apoptosis regulators during BTZ treatment was influenced by the absence of IFITM1. For the first time, the study demonstrated that the growth of MM cells promoted by insulin signalling is dependent on the presence of IFITM1. This finding suggests that IFITM1 may act as a downstream effector translating INSR activation into oncogenic behaviour in MM.

IFITM1 is important for immunity, anti‐viral activity, and cellular functions such as adhesion and proliferation [24]. IFITM1 forms a complex with the B cell receptor [25] and is involved in inflammatory diseases such as inflammatory bowel disease [26]. IFITM1 promotes homotypic adhesion of lymphocytes and inhibits proliferation of the B cell lineage [27, 28]. In addition, IFITM1 has been implicated in the migration, invasion, and proliferation of cancer cells [29, 30]; however, IFITM1 can also inhibit tumour cell growth [31]. The present study attempted to clarify the significance of the IFITM1 gene expression of malignant PCs induced by insulin signalling and to characterise IFITM1 gene regulation in the context of MM progression and treatment resistance. IFITM1 expression was previously demonstrated to be highly expressed in malignant PCs. Normal BM PC expression of IFITM1 is low. This suggests that IFITM1 expression is predominantly, if not exclusively, associated with MM. IFITM1, however, was not of prognostic relevance [32]. Our clinical data revealed IFITM1 expression significantly increased in symptomatic MM compared to MGUS and SMM. Furthermore, the five‐year PFS was significantly lower in the high IFITM1 group compared with the low IFITM1 group. We hypothesise that the prognostic significance of the highly expressed IFITM1 gene in symptomatic MM designates molecules that deserve further examination in the context of MM biology.

The INSR ligands insulin and IGF‐II are major growth factors in MM [33]. Previous studies identified the roles of insulin and IGF‐II as both growth and survival factors in MM. In vivo and in vitro studies proved that INSR ligands increase anti‐apoptotic protein expression, decrease pro‐apoptotic protein expression, and play a role in drug resistance [34, 35, 36]. Our results demonstrated that insulin increases MM cell proliferation and IFITM1 expression. Insulin induces cells to transition from G1 to S phase and to induce expected expression levels of pro‐ and anti‐apoptotic proteins. Mechanistically, insulin activated the mTOR and Akt signalling cascade, reflecting a metabolic shift toward an anabolic and proliferative state. These signalling changes were markedly diminished when IFITM1 was silenced, indicating that IFITM1 is required to sustain insulin‐driven activation of mTOR and Akt pathways. We also showed that the cytotoxic effect of BTZ, an important anti‐myeloma drug, could be mitigated by insulin according to the presence or absence of IFITM1. When IFITM1 is suppressed, cell viability is significantly decreased. Under these conditions, cell cycle arrest at the G1/S phase transition is significantly increased, and cell cycle progression through the S phase is significantly reduced [37]. These results suggest that IFITM1 may be critical for the growth and/or division of MM cells. Understanding the interaction between insulin signalling and IFITM1 may have implications for developing strategies to modulate the efficacy of BTZ in the treatment of MM, particularly in the context of insulin and IFITM1 expression. This study has some limitations. Our data were obtained only from in vitro studies. Further experiments using in vivo models are necessary to validate our findings and provide a more comprehensive understanding of the role of INSR signalling in regulating IFITM1 expression in MM. Additionally, mechanistic studies are needed to elucidate the specific pathways through which INSR signalling influences IFITM1 expression. By addressing these limitations, future studies can build upon our work and contribute to the development of novel therapeutic approaches for MM. Further research studies are also needed to explore the underlying mechanisms and potential therapeutic applications.

In conclusion, our study revealed that the expression of INSR and IFITM1 in malignant PCs increased as the disease advanced in MM patients. In particular, insulin signalling‐inducible IFITM1 expression promotes bortezomib resistance with significance in predicting prognosis. These findings suggest that IFITM1 may serve as a potential prognostic marker as well as treatment target in MM.

## Author Contributions

**Ji‐Young Lim:** conceptualization (equal), data curation (equal), formal analysis (equal), funding acquisition (equal), investigation (equal), methodology (equal), project administration (equal), software (equal), validation (equal), visualization (equal), writing – original draft (equal). **Yeojin Kim:** formal analysis (equal), methodology (equal), project administration (equal), software (equal), validation (equal), visualization (equal). **Sung‐Soo Park:** data curation (equal), formal analysis (equal), methodology (equal). **Jungyeon Lee:** data curation (equal), formal analysis (equal), methodology (equal), resources (equal). **Chang‐Ki Min:** funding acquisition (equal), supervision (equal), writing – review and editing (equal).

## Funding

This work was supported by the National Research Foundation of Korea (NRF) grant (2021R1A2C2093566, RS‐2023‐00212496).

## Conflicts of Interest

The authors declare no conflicts of interest.

## Supporting information


**Figure S1:** Time‐course analysis of INSR ligand effects on BTZ‐induced apoptosis in MM cells.
RPMI8226 and U266 cells were treated with BTZ in the presence or absence of insulin or IGF‐II for 24, 48, and 72 h. Representative overlaid histograms illustrate apoptotic cell fractions across different time points. The data are presented as mean ± SEM of three independent experiments. ***p < 0.001 (two‐way ANOVA).

**Figure S2:** Time‐course analysis of UNSR ligand effects on BTZ‐induced migration suppression in MM cells.
RPMI8226 and U266 cells were treated with BTZ in the presence or absence of insulin or IGF‐II for 6, 12, and 24 h. Cell migration was evaluated using Transwell assays and visualised by crystal violet staining. Representative overlaid histograms illustrate apoptotic cell fractions across different time points. The data are presented as mean ± SEM of three independent experiments. ***p < 0.001 (two‐way ANOVA).

## Data Availability

The data that support the findings of this study are available from the corresponding author upon reasonable request.
