# Supplementary figures and images for: Insulin Signalling‐Inducible IFITM1 Promotes Multiple Myeloma Progression and Bortezomib Resistance
Source: J Cell Mol Med. 2026 Jun 12;30(11):e71183. doi: 10.1111/jcmm.71183 (PMC13263400; doi:10.1111/jcmm.71183)

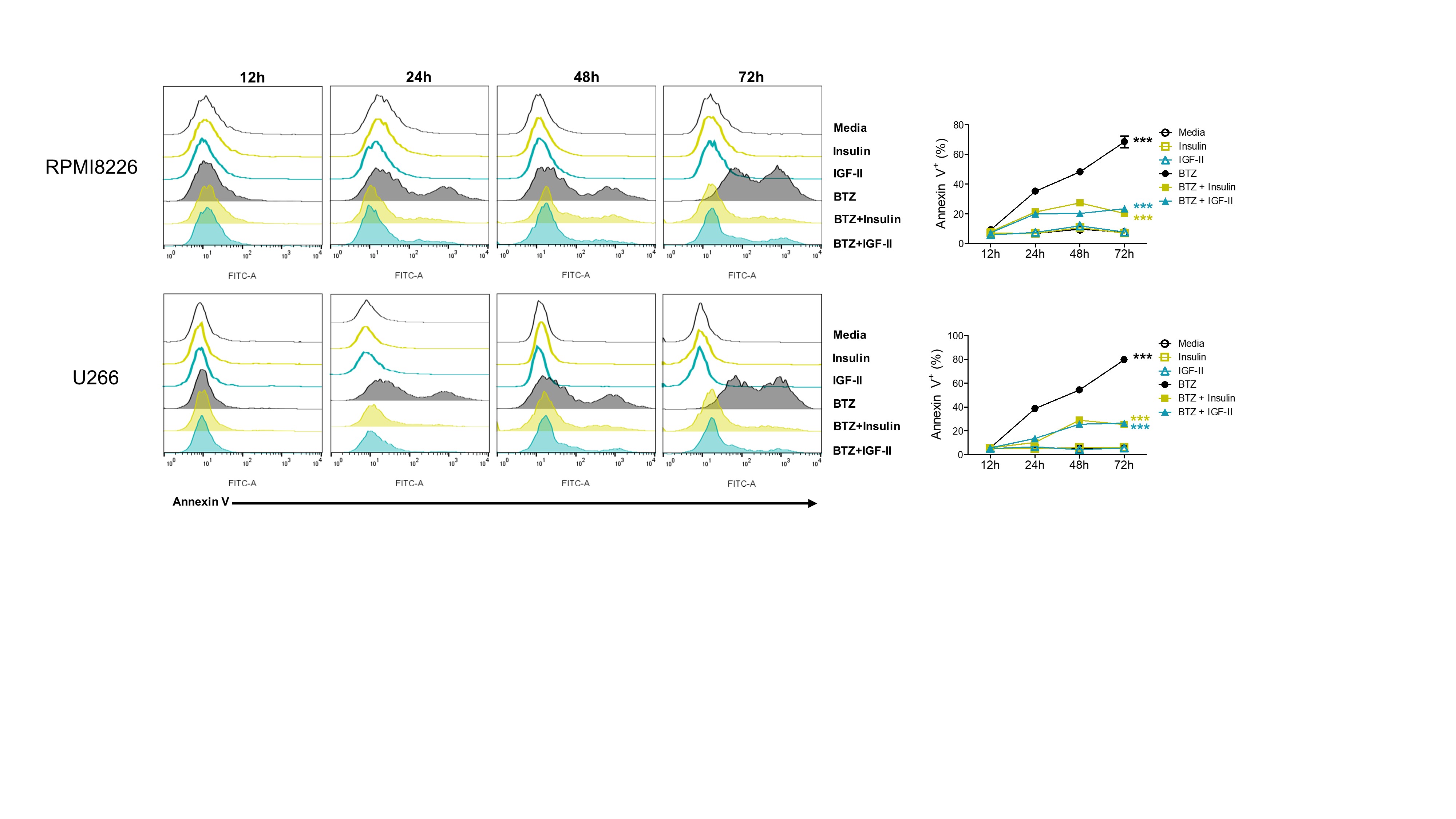

Supplement: Supplementary file 1 — Figure S1: Time‐course analysis of INSR ligand effects on BTZ‐induced apoptosis in MM cells. RPMI8226 and U266 cells were treated with BTZ in the presence or absence of insulin or IGF‐II for 24, 48, and 72 h. Representative overlaid histograms illustrate apoptotic cell fractions across different time points. The data are presented as mean ± SEM of three independent experiments. ***p < 0.001 (two‐way ANOVA). [file JCMM-30-e71183-s001.jpg]

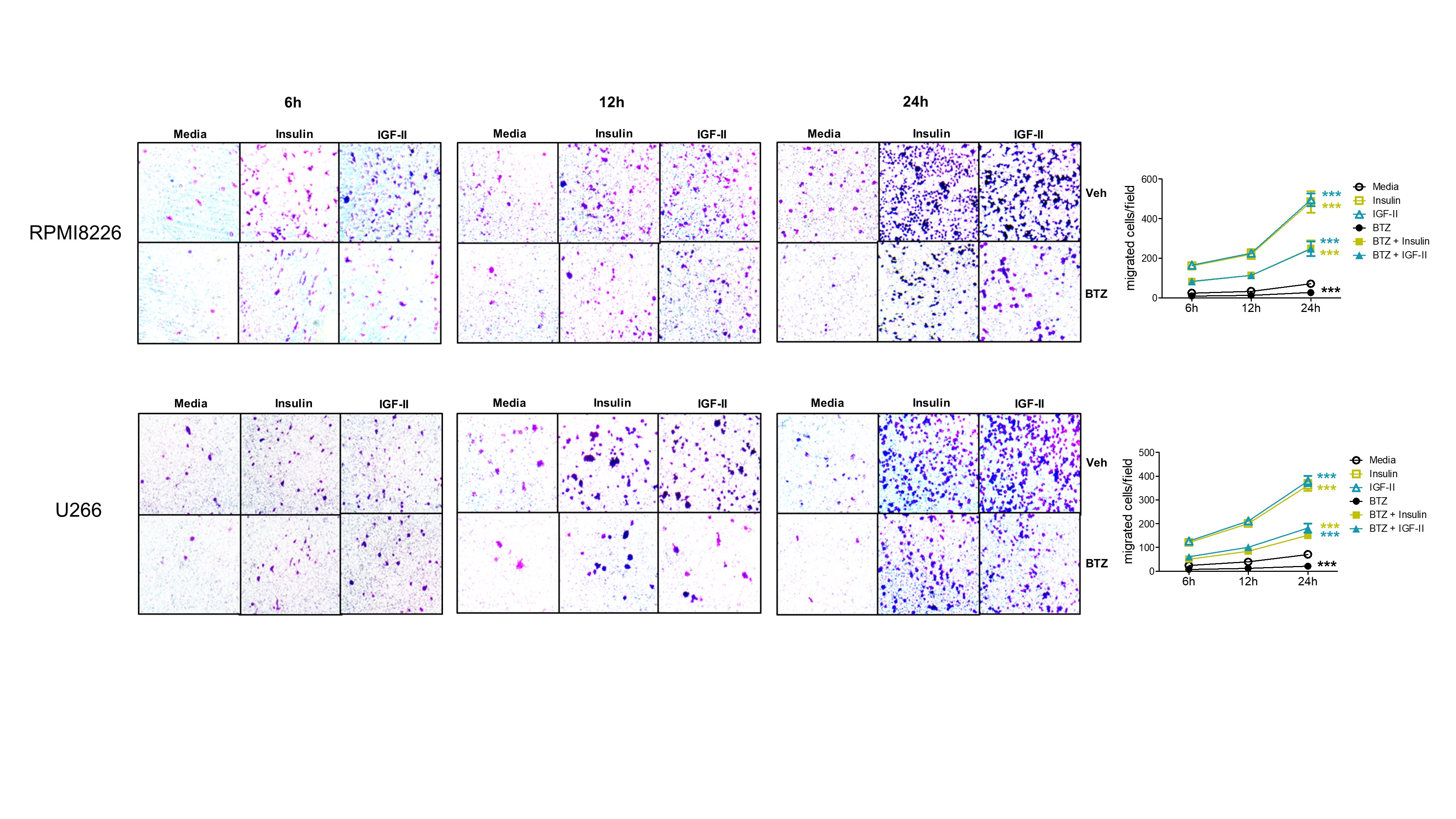

Supplement: Supplementary file 2 — Figure S2: Time‐course analysis of UNSR ligand effects on BTZ‐induced migration suppression in MM cells. RPMI8226 and U266 cells were treated with BTZ in the presence or absence of insulin or IGF‐II for 6, 12, and 24 h. Cell migration was evaluated using Transwell assays and visualised by crystal violet staining. Representative overlaid histograms illustrate apoptotic cell fractions across different time points. The data are presented as mean ± SEM of three independent experiments. ***p < 0.001 (two‐way ANOVA). [file JCMM-30-e71183-s002.jpg]
